# The frequency of asthma exacerbations and healthcare utilization in patients with asthma from the UK and USA

**DOI:** 10.1186/s12890-017-0409-3

**Published:** 2017-04-27

**Authors:** Robert Y. Suruki, Jonas B. Daugherty, Nada Boudiaf, Frank C. Albers

**Affiliations:** 1 Worldwide Epidemiology, GSK, Research Triangle Park, Durham, NC USA; 2 Value Outcomes and Epidemiology, PAREXEL International, Research Triangle Park, Durham, NC USA; 3 Worldwide Epidemiology, GSK, Stockley Park, Uxbridge, UK; 4 Respiratory Medical Franchise, GSK, Research Triangle Park, 5 Moore Drive, PO Box 13398, Durham, NC 27709 USA; 5 Present Address: UCB Biosciences, Epidemiology, Research Triangle Park, Durham, NC USA; 6 Present Address: Department of Pharmaceutical Outcomes and Policy, UNC Eshelman School of Pharmacy, University of North Carolina, Chapel Hill, NC USA; 7 Present Address: Chiltern International Ltd, Slough, Berkshire UK

**Keywords:** Exacerbation, Healthcare utilization, Electronic medical records, Claims database, Severe uncontrolled asthma, Severe eosinophilic asthma

## Abstract

**Background:**

Asthma exacerbations are frequent in patients with severe disease. This report describes results from two retrospective cohort studies describing exacerbation frequency and risk, emergency department (ED)/hospital re-admissions, and asthma-related costs by asthma severity in the US and UK.

**Methods:**

Patients with asthma in the US-based Clinformatics™ DataMart Multiplan IMPACT (2010–2011; WEUSKOP7048) and the UK-based Clinical Practice Research Datalink (2009–2011; WEUSKOP7092) databases were categorized by disease severity (Global Initiative for Asthma [GINA]; Step and exacerbation history) during the 12 months pre-asthma medical code (index date). Outcomes included: frequency of exacerbations (asthma-related ED visit, hospitalization, or oral corticosteroid use with an asthma medical code recorded within ±2 weeks) 12 months post-index, asthma-related ED visits/hospitalization, and asthma-related costs 30 days post-index. Risk of a subsequent exacerbation was determined by proportional hazard model.

**Results:**

Of the 222,817 and 211,807 patients with asthma included from the US and UK databases, respectively, 12.5 and 8.4% experienced ≥1 exacerbation during the follow-up period. Exacerbation frequency increased with disease severity. Among the 5,167 and 2,904 patients with an asthma-related ED visit/hospitalization in the US and UK databases, respectively, 9.2 and 4.7% had asthma-related re-admissions within 30 days. Asthma-related re-admission rates and costs increased with disease severity, approximately doubling between GINA Step 1 and 5 and in patients with ≥2 versus <2 exacerbations in the previous year. Risk of a subsequent exacerbation increased 32–35% for an exacerbation requiring ED visit/hospitalization versus oral corticosteroids.

**Conclusion:**

Increased disease severity was associated with higher exacerbation frequency, ED/hospitalization re-admission, costs and risk of subsequent exacerbation, indicating that these patients require high-intensity post-exacerbation management.

**Electronic supplementary material:**

The online version of this article (doi:10.1186/s12890-017-0409-3) contains supplementary material, which is available to authorized users.

## Background

Worldwide, 235 million people are estimated to suffer from asthma, [1] affecting 1–18% of the population in different countries [2]. Asthma is characterized by symptoms such as wheeze, chest tightness and shortness of breath, which can vary in intensity over time [2]. Exacerbations of asthma are acute or sub-acute episodes of increased symptoms and deteriorations in lung function, requiring an intensification of treatment [2]. Exacerbations occur across the spectrum of asthma severity but are more frequent in patients with severe disease and place a great burden on healthcare systems and patients [2–4].

Guidelines highlight the importance of using preventative therapies to reduce the risk and frequency of asthma exacerbations [2]. In patients with mild-to-moderate asthma, exacerbation frequency can be reduced by maintenance controller therapies such as inhaled corticosteroids (ICS) combined with long-acting β_2_-agonists (LABA) [2, 5–8]. However, in patients with severe asthma and persistent symptoms, a 5-year study has shown that exacerbation frequency remains relatively unchanged, despite intensive therapy with ICS/LABA for a minimum of 4 months per year [9]. Small-scale observational and database studies have demonstrated that the risk of a subsequent exacerbation is increased in patients with a history of frequent events and in patients with eosinophilic inflammation [9–11]. Healthcare resource utilization (HRU) is reported to be greatest in patients with severe asthma and a history of previous exacerbations, with exacerbations requiring oral corticosteroid (OCS) treatment, and emergency department (ED) visits or hospitalization documented as posing the greatest burden to healthcare systems [2, 12–15]. Additionally, the average direct costs per patient are reported to be approximately twice as high in patients with severe uncontrolled asthma compared with those that have non-severe uncontrolled asthma [16].

To improve disease management, there is a need to better characterize the burden of asthma exacerbations by disease severity. This report describes the results from two large retrospective cohort studies from the US and UK, which aimed to characterize the overall asthma exacerbation rates, their subsequent reoccurrence, associated HRU, and costs by asthma severity.

## Methods

### Study design

This report presents the results of two retrospective cohort studies of patients with asthma. The US study (GSK ID: WEUSKOP7048) identified patients from the US-based Clinformatics™ DataMart Multiplan (IMPACT) database (OptumInsight Life Sciences Inc., Eden Prairie, MN) (January 1, 2010–December 31, 2011). The UK study (GSK ID: WEUSKOP7092) identified patients from the UK-based Clinical Practice Research Datalink (CPRD; January 1, 2009–December 31, 2011). The index date was defined as the date of the first recorded asthma medical code during the study periods. In both databases, asthma severity and exacerbation history were determined during the 3-month and 12-month periods, respectively, prior to and including the study index date. During the 12-month period following asthma index date, the occurrence of exacerbations, medication use, and HRU associated with asthma exacerbations were determined in both the US and UK. Healthcare cost associated with asthma exacerbations was determined in the US only.

### Patients

Patients were identified in respective US and UK databases using the following inclusion criteria: ≥12 years of age, an asthma medical code (US: International Classification of Diseases, 9th Revision: 493.xx; UK: CPRD medical codes/Read codes) during the study period; at least one asthma medication (short-acting β_2_-agonists [SABA], short-acting muscarinic antagonist [USA only], ICS, ICS/LABA combination products, leukotriene receptor antagonist [LTRA], mast cell stabilizers, methylxanthines, or anticholinergics [long-acting muscarinic antagonist]) prescribed during the 3 months (US) or 30 days (UK) prior to the index date (inclusive); enrollment in respective databases for the 12 months before and after the index date. Exclusion criteria in both databases were: patients with a chronic obstructive pulmonary disease or cystic fibrosis diagnosis. In the US database, patients with a diagnosis for lung cancer were also excluded. Where available, UK database patient records in the CPRD were linked to the Hospital Episode Statistics (HES) data warehouse, containing records of all National Health Service hospital admissions and outpatient appointments.

### Outcomes and assessments

The frequency of ≥1 or ≥2 exacerbations in patients and the mean annual asthma exacerbation rate was assessed during the 12 months after the index date. Exacerbations were defined as a worsening of asthma requiring an ED/hospital admission or OCS treatment (US study: any OCS use equivalent to 20 mg/day prednisone for 3–28 days with an asthma medical code recorded within 2 weeks of the OCS prescription; UK: any OCS prescription within 2 weeks of an asthma medical code).

Exacerbations occurring within 7 days of the end date of an OCS prescription, ED admission, hospital discharge, or HES spell (healthcare episodes where a patient may have received care under more than one consultant within a hospital provider; UK only) were considered part of the same exacerbation [17]. Total all-cause and asthma-related ED/hospital re-admissions during the 30-, 60- and 90-day periods following a previous index asthma-related ED/hospital admission were also determined. The frequency of total HRU (US: inpatient admissions, ED visits, physician office visits, and other outpatient visits; UK: hospital admissions recorded by HES linked to CPRD, ED visits, general practice surgery visits, nurse visits, and telephone calls) was assessed during the 30-day period after an exacerbation. Asthma medication utilization (SABA, LTRA, ICS, ICS/LABA, OCS, anti-immunoglobulin E therapy [omalizumab], and theophylline and its derivatives]) during the 45-day period prior (to allow for gaps in prescriptions) and 30-day period after an asthma exacerbation was also assessed (non-mutually-exclusive categories). In the US study only, total-related costs (pharmacy, laboratory, and medical) and asthma-related costs (inpatient admissions, ED visits, outpatient visits, and pharmacy/medication costs) were determined from standardized insurance provider pricing and assessed during the 30-day period (inclusive) after an exacerbation.

### Covariates

Covariates assessed as potential confounders during the pre-index period included asthma severity, patient demographics (age, gender, atopic status) and exacerbation history. Asthma severity was assessed by medication use during the 90 days before the index date and categorized by Global Initiative for Asthma (GINA) or British Thoracic Society (BTS) Step in accordance with respective guidelines [2, 18]; separate medications were required to have a start date within 30 days of each other to be considered part of the same treatment regimen. Severity was also categorized by OCS and SABA prescription fills during the 12-month pre-index period, assessed by the modified Leidy category as defined by Wells et al. (2012) [2, 19]. Exacerbation history was determined from the 12-month period before the index date and presented as a categorical variable (number of exacerbations: 0, 1, 2, 3, ≥4) and binary variable (<2 or ≥2 exacerbations). Patients with ‘severe uncontrolled asthma’ were defined as patients at GINA Step 4 or 5 with ≥2 exacerbations in the previous 12 months. Patients with ‘severe uncontrolled eosinophilic asthma’ were defined as fulfilling the criteria for ‘severe uncontrolled asthma’ and had blood eosinophil counts ≥300 cells/μl at baseline. Atopic status was determined by the presence of an allergic rhinitis medical code during the baseline period. Exacerbation season was determined as: spring (March–May), summer (June–August), fall (September–November), and winter (December–February).

### Statistical analysis

Demographic characteristics were reported as count (%) for categorical variables and mean (standard deviation) for continuous variables. Mean annual asthma exacerbation rates were calculated using a simple unadjusted negative binomial regression model and described with 95% confidence intervals. The association between the severity of asthma exacerbations and the risk of subsequent exacerbations was analyzed using a multi-variable Cox regression model, incorporating covariates with a *p*-value >0.10 and potential confounder variables with a *p*-value <0.10. Age, gender, asthma severity, and exacerbation history were also included. For ICS claims where the number of days treatment supply was missing or was <15 days, a value of 30 days was imputed; for ICS/LABA, LTRA or methylxanthines claims where supply length was missing or <30 days, a value of 30 days was imputed.

## Results

### Patients

In total, data from 222,817 and 211,807 patients in the US and UK databases, respectively, were included in the study (Table 1). Overall, patients in the US had a lower average age than the UK. During the pre-index period, patients in the US were less likely to be classified at GINA Step 4/5 (moderate severe) and had a higher mean annual exacerbation rate compared with the UK.

**Table 1:** Patient demographics and baseline characteristics during the 12 months before the index date

		US database (*N* = 222,817)			UK database (*N* = 211,807)		
		Patients (classified during the pre-index period) *N* (%)^a^	Patients with ≥1 exacerbation 12-months post-index *N* (%)^a^	Patients with ≥2 asthma-exacerbations 12-months post-index *N* (%)^a^	Study population (classified during the pre-index period) *N* (%)^a^	Patients with ≥1 exacerbation 12-months post-index *N* (%)^a^	Patients with ≥2 asthma-exacerbations 12-months post-index *N* (%)^a^
Overall		222,817 (100.0)	27,865 (12.5)	5,560 (2.5)	211,807 (100.0)	17,785 (8.4)	3,592 (1.7)
Age (years), mean (SD)		38.0 (16.6)	38.2 (16.4)	38.9 (16.2)	45.0 (20.1)	47.5 (19.2)	47.9 (19.1)
Gender, female		134,123 (60.2)	17,829 (64.0)	3,676 (66.1)	122,577 (57.9)	11,889 (66.9)	2,522 (70.2)
GINA Step	Step 1	103,415 (46.4)	11,513 (41.3)	2,057 (37.0)	37,438 (17.7)	2,019 (11.4)	300 (8.4)
	Step 2	36,616 (16.4)	4,140 (14.9)	780 (14.0)	35,488 (16.8)	2,075 (11.8)	302 (8.4)
	Step 3	38,497 (17.3)	4,722 (16.9)	864 (15.5)	78,863 (37.2)	5,575 (31.4)	913 (25.4)
	Step 4	36,039 (16.2)	5,155 (18.5)	1,109 (19.9)	49,169 (23.2)	4,811 (27.1)	952 (26.5)
	Step 5	7,736 (3.5)	2,288 (8.2)	744 (13.4)	10,291 (4.9)	3,252 (18.3)	1,117 (31.3)
	Not classifiable/none	514 (0.2)	47 (0.2)	6 (0.1)	558 (0.3)	53 (0.3)	8 (0.2)
Exacerbations during the 12-month pre-index period	0	180,830 (81.2)	16,830 (60.4)	2,498 (44.9)	182,712 (86.3)	10,853 (61.0)	1,500 (41.8)
	1	36,966 (16.6)	8,572 (30.8)	2,010 (36.2)	25,129 (11.9)	5,096 (28.7)	1,255 (34.9)
	2	3,758 (1.7)	1,629 (5.8)	603 (10.8)	2,991 (1.4)	1,213 (6.8)	443 (12.3)
	3	833 (0.4)	505 (1.8)	232 (4.2)	633 (0.3)	361 (2.0)	194 (5.4)
	≥4	430 (0.2)	329 (1.2)	217 (3.9)	342 (0.2)	262 (1.5)	200 (5.6)
Exacerbation rate per patient year, mean (95% CI)		0.220 (0.218, 0.222)	0.536 (0.528, 0.545)	0.902 (0.878, 0.928)	0.164 (0.162, 0.165)	0.560 (0.547, 0.573)	1.062 (1.016, 1.110)
Severe uncontrolled asthma^b^	Yes	2,059 (0.9)	1,106 (4.0)	510 (9.2)	2,552 (1.2)	1,321 (7.4)	655 (18.2)
	No	220,758 (99.1)	26,759 (96.0)	5,050 (90.8)	209,255 (98.8)	16,464 (92.6)	2,937 (81.8)
Severe uncontrolled eosinophilic asthma^c^	Yes	114 (0.5)	69 (2.2)	27 (4.4)	357 (0.7)	217 (4.1)	126 (10.8)
	No	25,131 (99.5)	3,053 (97.8)	580 (95.6)	50,089 (99.3)	5,118 (95.9)	1,045 (89.2)

Further characteristics including age range, Leidy category, and proportion of days covered are listed in Additional file 1: Table S1; ^a^proportions calculated as a percentage of the overall population in each column; ^b^defined as patients at GINA Step 4 or 5 with ≥2 exacerbations in the previous 12 months; ^c^patients with severe uncontrolled asthma criteria and blood eosinophil counts ≥300 cells/μl at baseline

*CI* confidence interval, *GINA* Global Initiative for Asthma, *SD* standard deviation

During the 12-month follow-up period, 27,865 (12.5%) and 5,560 (2.5%) patients in the US, and 17,785 (8.4%) and 3,592 (1.7%) of patients in the UK experienced ≥1 and ≥2 exacerbations, respectively (Table 1). Compared with patients with ≥1 exacerbations, patients with ≥2 exacerbations tended to have more severe disease defined as higher GINA Step, a history of more frequent exacerbations or severe uncontrolled and severe uncontrolled eosinophilic asthma (Table 1). Further patient details are available in Additional file 1: Table S1.

### Exacerbation frequency

**Fig. 1:**
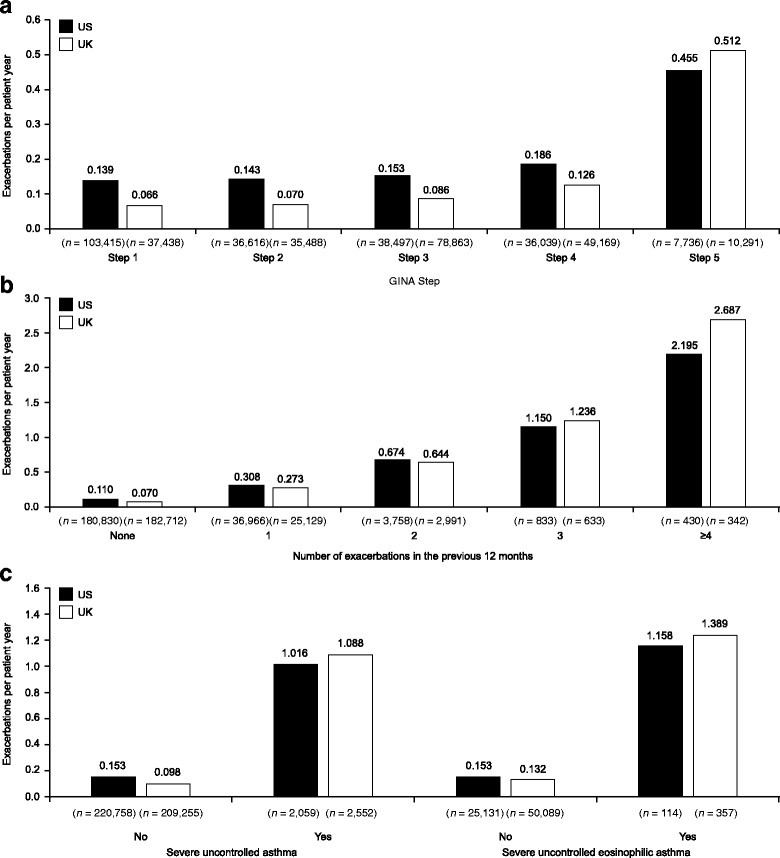
Exacerbation rates during the follow-up period by **a** GINA category; **b** exacerbation history, and **c** severe and severe uncontrolled eosinophilic asthma. Severe asthma defined as patients at GINA Step 4 or 5 with ≥2 exacerbations in the previous 12 months; Severe eosinophilic asthma defined as patients with severe uncontrolled asthma criteria and blood eosinophil counts ≥300 cells/μl at baseline. GINA, Global Initiative for Asthma

The overall mean annual exacerbation rates per patient during the 12-month period after the index date in the US and UK were 0.16/year and 0.11/year, respectively (Table 2). Mean annual exacerbation frequency increased with indicators of disease severity (GINA Step, severe uncontrolled and uncontrolled eosinophilic asthma status) and with historical exacerbation frequency (Table 2; Fig. 1a–c). Exacerbations were more frequent in females, in atopic patients, and their frequency increased with Leidy category (Additional file 1: Table S2). Mean annual exacerbation rate trends during the 12-month follow-up period were broadly consistent across covariates in both the US and UK, although overall mean annual exacerbation rates were higher in the US.

**Table 2:** Mean annual exacerbation rate per patient year in the 12 months post-index date by covariate

		US database (*N* = 222,817) Rate (95% CI)	UK database (*N* = 211,807) Rate (95% CI)
Overall		0.161 (0.159, 0.163)	0.110 (0.109, 0.111)
GINA Step	Step 1	0.139 (0.136, 0.142)	0.066 (0.063, 0.069)
	Step 2	0.143 (0.138, 0.147)	0.070 (0.067, 0.073)
	Step 3	0.153 (0.148, 0.158)	0.086 (0.084, 0.089)
	Step 4	0.186 (0.181, 0.191)	0.126 (0.123, 0.130)
	Step 5	0.455 (0.434, 0.478)	0.512 (0.490, 0.536)
	Not classifiable/none	0.105 (0.078, 0.142)	0.113 (0.084, 0.150)
Exacerbations during the 12-month pre-index period	0	0.110 (0.109, 0.112)	0.070 (0.069, 0.071)
	1	0.308 (0.301, 0.315)	0.273 (0.265, 0.282)
	2	0.674 (0.638, 0.712)	0.644 (0.601, 0.690)
	3	1.150 (1.036, 1.276)	1.236 (1.080, 1.414)
	≥4	2.195 (1.925, 2.504)	2.687 (2.269, 3.183)
Severe uncontrolled asthma^a^	Yes	1.016 (0.939, 1.099)	1.088 (1.002, 1.181)
	No	0.153 (0.151, 0.155)	0.098 (0.096, 0.100)
Severe uncontrolled eosinophilic asthma^b^	Yes	1.158 (0.828, 1.619)	1.389 (1.127, 1.712)
	No	0.153 (0.148, 0.159)	0.132 (0.128, 0.136)

^a^Defined as patients at GINA Step 4 or 5 with ≥2 exacerbations in the previous 12 months; ^b^patients with severe uncontrolled asthma criteria and blood eosinophil counts ≥300 cells/μl at baseline. *CI* confidence interval, *GINA* Global Initiative for Asthma

### Risk of a subsequent asthma exacerbation

In an adjusted proportional hazard model, patients who experienced an exacerbation requiring an ED/hospital admission had a 35 and 32% greater risk of a subsequent exacerbation during the 12 months after the index date compared with patients experiencing exacerbations only requiring OCS in the US and UK, respectively (Table 3). Patients with the most severe disease as assessed by GINA (US) or BTS (UK) Step demonstrated the highest risk of a subsequent exacerbation during the 12 months after the index date. In particular, the adjusted risk of a subsequent exacerbation was increased by 44% for patients at GINA Step 5 compared with at Step 1–3 (US) and by 87% at BTS Step 5 versus Step 1 (UK). Other factors associated with an increased risk of a subsequent exacerbation were ≥1 exacerbation in the previous year (assessed in the UK only) (Table 3), female gender, a higher Leidy category, and atopic status (assessed in the UK only) (Additional file 1: Table S3).

**Table 3:** Risk of a subsequent exacerbation of patients with ≥1 exacerbation during the 12-month after a previous exacerbation

		US database	UK database
		*N* = 27,865	*N* = 17,785
	Covariates	HR (95% CI)^a^	HR (95% CI)^b^
Exacerbation type	OCS only	Ref.	Ref.
	ED/hospitalization	1.35 (1.26, 1.44)	1.32 (1.18, 1.47)^*^
Exacerbations during the 12-month pre-index period	0	NR	Ref.
	≥1	NR	1.90 (1.75, 2.05)^*^
GINA (US only) or BTS (UK only) Treatment Steps^c^	Step 1	Steps 1–3 combined: Ref.	Ref.
	Step 2		1.02 (0.90, 1.17)
	Step 3		1.35 (1.19, 1.53)^*^
	Step 4	1.26 (1.18, 1.35)	1.44 (1.25, 1.65)^*^
	Step 5	1.44 (1.32, 1.56)	1.87 (1.64, 2.14)^*^
	Not classifiable/none	0.76 (0.34, 1.69)	1.36 (0.85, 2.19)

*p < 0.0001

^a^Multivariable proportional hazards model with the following covariates: exacerbation type, gender, GINA Step, Leidy category

^b^multivariable proportional hazards model with the following covariates: exacerbation type, gender, BTS Step, atopy, Leidy category and exacerbation history; ^c^GINA failed the proportionality test so was removed from the UK database

*BTS* British Thoracic Society, *CI* confidence interval, *ED* emergency department, *GINA* Global Initiative for Asthma, *HR* hazard ratio, *NR* not referenced, *OCS* oral corticosteroids

## HRU

### ED/hospital re-admission

**Fig. 2:**
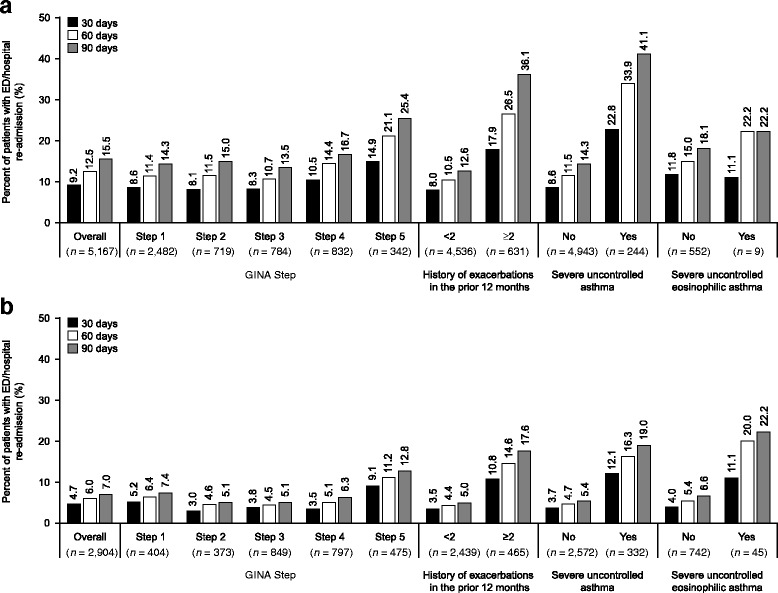
Proportion of patients with an asthma-related ED/hospital re-admission after being discharged for an asthma-related ED/hospital admission within the **a** US database and **b** UK database. Severe asthma defined as patients at GINA Step 4 or 5 with ≥2 exacerbations in the previous 12 months; Severe eosinophilic asthma defined as patients with severe uncontrolled asthma criteria and blood eosinophil counts ≥300 cells/μl at baseline. ED, emergency department; GINA, Global Initiative for Asthma

**Fig. 3:**
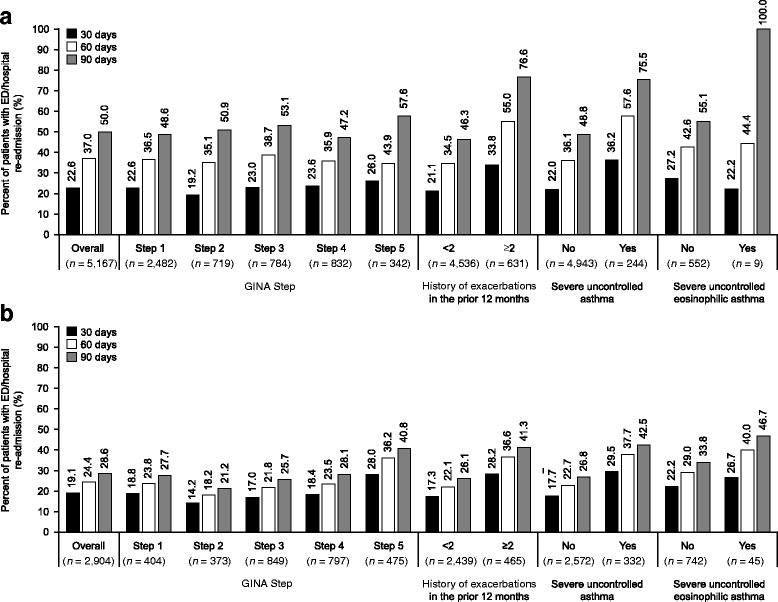
Proportion of patients with an all-cause ED/hospital re-admission following an index asthma-related ED/hospital admission within the **a** US database and **b** UK database. Severe asthma defined as patients at GINA Step 4 or 5 with ≥2 exacerbations in the previous 12 months; Severe eosinophilic asthma defined as patients with severe uncontrolled asthma criteria and blood eosinophil counts ≥300 cells/μl at baseline. ED, emergency department; GINA, Global Initiative for Asthma

In the 30-day period following discharge for an asthma-related ED/hospital visit, asthma-related ED/hospital re-admissions occurred in 9.2 and 4.7% of patients and all-cause ED/hospital re-admissions occurred in 22.6 and 19.1% of patients in the US and UK, respectively. Both asthma-related and all-cause ED/hospital patient re-admissions within 30 days were highest in patients at GINA Step 5 versus Step 1, a history of ≥2 versus <2 exacerbations and severe uncontrolled asthma versus non-severe asthma. Asthma-related and all-cause re-admission in patients with severe uncontrolled eosinophilic asthma versus non-severe asthma were lower in the US but higher in the UK (Fig. 2a and b; Fig. 3a and b). Similar trends were seen for the 60- and 90-day periods (Fig. 2a and b; Fig. 3a and b). Results for ED/hospital re-admissions by age, gender, Leidy category, and atopic status are shown in Additional file 1: Table S4 and S5.

### Total HRU after an exacerbation

The overall mean frequency of total HRU during the 30-day period after an exacerbation was 1.35 and 2.49 interactions/patient in the US and UK, respectively. Total HRU frequency was highest in patients at GINA Step 5 versus 1 and a history of ≥2 versus <2 exacerbations during the pre-index period (Additional file 1: Table S6). Similarly, the total HRU frequency was higher in patients with severe uncontrolled asthma, severe uncontrolled eosinophilic asthma and with atopic asthma compared with those without (Additional file 1: Table S6). Additionally, total HRU was similar across age groups in the US, but increased with age in the UK (Additional file 1: Table S6). Overall, the frequency of total HRU was lower in the US than the UK across all covariates.

### Medication HRU before and after an exacerbation

In the 45-day period before an exacerbation, 20.9 and 42.8% of patients received ICS/LABA, and 21.3 and 22.7% of patients received OCS in the US and UK, respectively. During the 30-day period after an exacerbation, the most commonly used medications were SABAs followed by ICS/LABA and LTRA in the US and SABAs, ICS/LABA and OCS in the UK (Additional file 1: Table S7). ICS + LABA and OCS use increased with age and markers of disease severity including exacerbation history, severe uncontrolled asthma status, severe uncontrolled eosinophilic asthma status, and atopy (Additional file 1: Table S7). Medication use also increased with GINA Step and Leidy category, both of which are determined by medication use. Overall, medication use, including ICS/LABA and OCS, tended to be lower in the US than the UK (Additional file 1: Table S7).

### Healthcare costs after an exacerbation (assessed in US database only)

In the US, during the 30-day period after an exacerbation including the index date, the mean all-cause total insurance provider healthcare cost per exacerbation was $1,368 (Table 4). Total all-cause costs per exacerbation increased with disease severity and with a history of ≥2 versus <2 exacerbations/year. Of all the parameters assessed, costs were highest for patients with severe uncontrolled eosinophilic asthma, reaching $2,040 per exacerbation in the 30 days after an exacerbation including the index date. When the analysis was restricted to just asthma-related healthcare costs, the mean total healthcare cost per exacerbation was $520 (Table 4). As with all-cause total healthcare cost per exacerbation, total asthma-related costs increased with disease severity and in patients a history of ≥2 versus <2 exacerbations/year and were highest for patients with severe uncontrolled eosinophilic asthma (Table 4). Further details of all-cause and asthma-related healthcare costs are shown in Additional file 1: Table S8 and S9.

**Table 4:** Mean healthcare cost per exacerbation in the 30 days after an exacerbation (US database only)

		Total all-cause ($)	Total asthma-related ($)
		mean (SD)	mean (SD)
Overall		1,368 (3,797)	520 (981)
GINA Step	Step 1	1,265 (3,981)	446 (908)
	Step 2	1,376 (3,978)	465 (874)
	Step 3	1,253 (3,242)	433 (782)
	Step 4	1,425 (3,817)	516 (828)
	Step 5	1,814 (3,614)	902 (1,533)
	Not classifiable/none	946 (1,628)	332 (377)
Exacerbations during the 12-month pre-index period	<2	1,344 (3,836)	477 (861)
	≥2	1,534 (3,516)	754 (1,451)
Severe uncontrolled asthma^a^	Yes	1,692 (3,233)	911 (1,563)
	No	1,347 (3,831)	486 (905)
Severe uncontrolled eosinophilic asthma^b^	Yes	2,040 (3,285)	1,073 (1,958)
	No	1,692 (3,934)	615 (1,248)

^a^Defined as patients at GINA Step 4 or 5 with ≥2 exacerbations in the previous 12 months; ^b^patients with severe uncontrolled asthma criteria and blood eosinophil counts ≥300 cells/μl at baseline

*GINA* Global Initiative for Asthma, *SD* standard deviation

## Discussion

In this report of two large database studies in the US and UK, each involving >200,000 patients with asthma, it was demonstrated that increased disease severity defined by GINA Step, medication use, and exacerbation history was associated with higher asthma exacerbation frequency. Furthermore, patients with an asthma-related exacerbation leading to ED/hospital re-admission had a 32–35% increased risk of a subsequent exacerbation during the 12 months after the index date in both countries and healthcare resource use within 30 days of an exacerbation was highest for patients with the most severe disease. While overall trends were similar between the US and UK, US patients demonstrated a higher exacerbation frequency, lower medication use, and higher ED/hospital re-admission rates compared with UK patients.

The trends demonstrated in both the US and UK are similar to previous studies. The smaller-scale TENOR study demonstrated that exacerbation frequency was higher in patients who had a recent history of an exacerbation compared with those who did not [10]. In a study of health insurance claims from approximately 2,500 patients with persistent asthma, the occurrence of an exacerbation in the previous year was found to increase the risk of a subsequent exacerbation nearly 8-fold [9]. Exacerbation severity has also been shown to increase subsequent exacerbation risk. The TENOR study demonstrated that the occurrence of a recent severe exacerbation requiring an ED/hospital admission increased the odds of a future exacerbation nearly 3-fold following adjustment for key covariates, compared with patients with exacerbations not requiring admissions [10]. In the current large database studies, following adjustment for confounding covariates, the risk of a subsequent exacerbation during the following year was increased by 35% (US) and 32% (UK) in patients who had a previous exacerbation requiring an ED/hospital visit compared with patients requiring only OCS treatment. Exacerbation risk has also been demonstrated to increase with eosinophilia [11]. Although in the current study eosinophil counts were not assessed as a risk factor, it was demonstrated that the proportion of patients experiencing ≥1 exacerbations during the follow-up period was higher in patients who had severe uncontrolled asthma (GINA Step 4/5 plus ≥2 exacerbations in the previous year) with eosinophil counts (≥300 cells/μl) than in patients who did not. The current study also demonstrated that females had a higher exacerbation risk than males, a result demonstrated in a previous study, [20] although not all studies are in agreement [21].

As expected, in both countries, the use of ICS/LABA and OCS increased with markers of disease severity including GINA Step, Leidy category, and exacerbation history, and was higher in patients with severe uncontrolled asthma or severe uncontrolled eosinophilic asthma status versus patients who had non-severe asthma. In the current study, despite the increased medication usage in patients with more severe disease, total HRU during the 30 days after an exacerbation was still highest in these patients. Reflecting this, a previous study demonstrated in patients with persistent asthma symptoms that despite high-intensity treatment, the number of asthma exacerbations over a 5-year period remained relatively unchanged [9]. Most strikingly, the proportion of patients with asthma-related ED/hospital re-admissions in the 30-day period following a previous ED/hospital admission approximately doubled between GINA Step 3 and Step 5. Similarly, a history of frequent exacerbations and a severe disease classification increased asthma-related re-admission rates, with up to 23% experiencing an asthma-related re-admission within 30 days of discharge. These trends are reflected in a previous small-scale US-based study, which demonstrated that the majority of ED visits were made by the most severe patients (≥6 ED visits in the previous year) and that markers of severity were independent predictors of high ED use [14]. Together, these results indicate that patients with severe disease are the most likely to require further healthcare utilization after an asthma exacerbation, suggesting that higher levels of step-up care and the use of novel drug classes may be needed [22].

In terms of healthcare costs in the US during the 30 days after an exacerbation, asthma-related costs were higher in patients with a greater history of exacerbations and categorized as having severe uncontrolled asthma or severe uncontrolled eosinophilic asthma compared with patients who did not meet these criteria. Specifically, patients with severe uncontrolled eosinophilic asthma, the asthma-related cost was $1,073 compared with $520 in the overall asthma population during the 30 days after an exacerbation. Similarly, an observational study by Zeiger et al. (2015) demonstrated that the annual asthma-related costs approximately doubled in patients with severe uncontrolled asthma compared with patients with non-severe uncontrolled asthma [16]. Broadly, the results of both studies are in accordance with other small-scale [13] and large-scale population studies [15].

Whilst the overall trends in both databases were similar, some differences were identified between the US and UK. In the UK, the exacerbation frequency for patients at GINA Step 1–3 was approximately half that of the US, despite the frequency of exacerbations being similar between the UK and US for GINA Step 4–5. This may be a reflection of the higher ICS/LABA and OCS use at these lower treatment steps in the UK compared with the US, since ICS/LABA use has been demonstrated to decrease exacerbation frequency [2, 5]. Asthma-related ED/hospital re-admission within 30 days was also higher in the US compared with the UK, which could also be related to medication use or differences in the access to primary care physicians in these countries.

The strength of these studies is that they sample data from a large number of patients (>400,000 across the US and UK), allowing for the characterization of exacerbation frequency and HRU across a wide range of asthma severities and provide insights into the differences between the two populations. However, there are also several limitations, some inherent to the retrospective database methodology. Disease severity was inferred by GINA or BTS Step rather than clinical measurements, which may have led to severity being incorrectly assigned due to fluctuations in physician prescribing behavior over time. Limitations that affect database comparability include differences in the type of data that the US (prescription claims) and UK (medication prescriptions) databases use, which may have led to an underestimated or overestimated medication use. In the US specifically, this may have contributed to an under-reporting of SABA use, as patients may not have required a new SABA prescription if the previous one had not been completely used. Further limitations that may have affected database comparability include: 1) the inclusion of lung cancer as an exclusion criteria in the US but not UK cohort (these data are not well captured and recorded in the CPRD); 2) lack of claims data in the UK CPRD versus US IMPACT database; 3) the inclusion of only patients with commercial health insurance in the US IMPACT versus UK CPRD database potentially reducing representation of the full asthmatic population; and 4) UK database patients were not required to be HES-linked, potentially resulting in the under-reporting of ED visits and HES spells specific for the treatment of exacerbations. This may have also prevented the accurate differentiation of exacerbation severity based on ED visit/hospitalization. However, as BTS and GINA guidelines both recommend treatment with OCS after ED/hospitalization discharge for an exacerbation, it is likely that most exacerbations were captured in the data sources sampled.

## Conclusions

Using data from >200,000 patients with asthma in both the US and UK, we demonstrated that the frequency and risk of asthma exacerbations, HRU, and healthcare costs (US only) all increased with disease severity. In particular, these outcomes increased in patients with higher GINA Step, a frequent history of exacerbations, and severe uncontrolled and severe uncontrolled eosinophilic asthma status. Interestingly, the results suggest a higher exacerbation frequency, lower medication use and higher ED/hospital re-admission rates in the US compared with the UK. Appropriate post-discharge medication, healthcare practitioner follow-up, and asthma management plans should be ensured for patients admitted to the ED or hospital for asthma. Patients with severe uncontrolled asthma warrant more attentive post-discharge clinical supervision given their increased risk of a recurrent ED/hospital re-admission.

## Additional file

Additional file 1: Tables S1–S9. Additional results for patient demographics and baseline characteristics, exacerbation rates, exacerbation risk, frequency of hospital re-admissions, healthcare utilization, medication utilization and healthcare costs. (DOC 304 kb)

## Abbreviations

BTS: British Thoracic Society
CI: Confidence interval
CPRD: Clinical Practice Research Datalink
ED: Emergency department
GINA: Global Initiative for Asthma
HES: Hospital Episode Statistics
HR: Hazard ratio
HRU: Healthcare resource utilization
ICS: Inhaled corticosteroid
IgE: Immunoglobulin E
LABA: Long-acting β_2_-agonist
LTRA: Leukotriene receptor antagonist
N/A: Not applicable
NR: Not referenced
OCS: Oral corticosteroid
SABA: Short-acting β_2_-agonist
SD: Standard deviation
