# Supplementary material for: The frequency of asthma exacerbations and healthcare utilization in patients with asthma from the UK and USA
Source: BMC Pulm Med. 2017 Apr 27;17:74. doi: 10.1186/s12890-017-0409-3 (PMC5406966; doi:10.1186/s12890-017-0409-3)
Supplement: Additional file 1: Tables S1–S9. — Additional results for patient demographics and baseline characteristics, exacerbation rates, exacerbation risk, frequency of hospital re-admissions, healthcare utilization, medication utilization and healthcare costs. (DOC 304 kb) [file 12890_2017_409_MOESM1_ESM.doc]

**Supplementary Tables**

**Table S1.** Patient demographics and baseline characteristics during the 12 months before the index date

|  | | | **US database**  **(N=222,817)** | | | **UK database**  **(N=211,807)** | | |
| --- | --- | --- | --- | --- | --- | --- | --- | --- |
|  | | | **Patients (classified during the pre-index period)**  **N (%)^a^** | **Patients  with ≥1 exacerbation  12-months  post-index**  **N (%)^a^** | **Patients  with ≥2 asthma-exacerbations 12-months  post-index**  **N (%)^a^** | **Study population (classified during the pre-index period)**  **N (%)^a^** | **Patients  with ≥1 exacerbation  12-months  post-index**  **N (%)^a^** | **Patients  with ≥2 asthma-exacerbations  12-months  post-index**  **N (%)^a^** |
| **N=** |  | | 222,817 | 27,865 | 5,560 | 211,807 | 17,785 | 3592 |
| **Age group, years** | 12–17 | | 39,413 (17.7) | 4,900 (17.6) | 923 (16.6) | 25,561 (12.1) | 1,502 (8.5) | 297 (8.3) |
|  | 18–34 | | 51,762 (23.2) | 6,035 (21.7) | 1,114 (20.0) | 42,492 (20.1) | 3,117 (17.5) | 603 (16.8) |
|  | 35–44 | | 43,662 (19.6) | 5,672 (20.4) | 1,168 (21.0) | 36,239 (17.1) | 3,122 (17.6) | 640 (17.8) |
|  | 45–54 | | 46,265 (20.8) | 6,133 (22.0) | 1,336 (24.0) | 36,269 (17.1) | 3,450 (19.4) | 711 (19.8) |
|  | 55–64 | | 34,161 (15.3) | 4,346 (15.6) | 860 (15.5) | 30,629 (14.5) | 2,850 (16.0) | 562 (15.7) |
|  | 65+ | | 7,554 (3.4) | 779 (2.8) | 159 (2.9) | - | - | - |
| **Leidy category** | | 1. Low | 98,450 (44.2) | 7,201 (25.8) | 879 (15.8) | 64,751 (30.6) | 2,941 (16.5) | 345 (9.6) |
|  |  | 2. Low-to-Moderate | 85,902 (38.6) | 11,187 (40.1) | 1,889 (34.0) | 101,496 (47.9) | 7,617 (42.8) | 1,220 (34.0) |
|  |  | 3. Moderate-to-Severe | 27,175 (12.2) | 5,699 (20.5) | 1,410 (25.4) | 44,833 (21.2) | 6,869 (38.6) | 1,853 (51.6) |
|  |  | 4. Severe | 11,290 (5.1) | 3,778 (13.6) | 1,382 (24.9) | 727 (0.3) | 358 (2.0) | 174 (4.8) |

^a^Proportions calculated as a percentage of the overall population in each column.

**Table S2.** Mean annual exacerbation rate per patient year in the 12 months post-index date by covariate

|  | | **US database**  **(N=222,817)**  **Rate (95% CI)** | | **UK database**  **(N=211,807)**  **Rate (95% CI)** |
| --- | --- | --- | --- | --- |
| **Age group, years** | 12–17 | 0.156 (0.152, 0.161) | 0.077 (0.073, 0.081) | |
|  | 18–34 | 0.147 (0.143, 0.151) | 0.096 (0.093, 0.100) | |
|  | 35–44 | 0.169 (0.165, 0.174) | 0.114 (0.110, 0.119) | |
|  | 45–54 | 0.174 (0.170, 0.179) | 0.125 (0.120, 0.130) | |
|  | 55–64 | 0.162 (0.157, 0.168) | 0.119 (0.115, 0.124) | |
|  | 65+ | 0.133 (0.124, 0.143) | 0.121 (0.116, 0.125) | |
| **Gender** | Female | 0.172 (0.169, 0.174) | 0.128 (0.126, 0.130) | |
|  | Male | 0.144 (0.141, 0.147) | 0.085 (0.083, 0.087) | |
| **Leidy category** | 1. Low | 0.084 (0.082, 0.086) | 0.052 (0.050, 0.054) | |
|  | 2. Low-to-Moderate | 0.159 (0.156, 0.162) | 0.092 (0.089, 0.094) | |
|  | 3. Moderate-to-Severe | 0.283 (0.275, 0.290) | 0.221 (0.216, 0.227) | |
|  | 4. Severe | 0.547 (0.528, 0.566) | 0.944 (0.807, 1.105) | |
| **Atopic** | Yes | 0.187 (0.183, 0.191) | 0.143 (0.134, 0.154) | |
|  | No | 0.147 (0.145, 0.149) | 0.108 (0.107, 0.110) | |

CI, confidence interval.

**Table S3.** Risk of a subsequent exacerbation of patients with ≥1 exacerbation during the 12 months after a previous exacerbation

|  |  | **US database** | **UK database** |
| --- | --- | --- | --- |
|  |  | N=27,865 | N=17,785 |
|  | **Covariates** | **HR (95% CI)^a^** | **HR (95% CI)^b^** |
| **Gender** | Male | Ref. | Ref. |
|  | Female | 1.09 (1.03, 1.15) | 1.13 (1.05, 1.21)^†^ |
| **Leidy category** | 1. Low  2. Low-to-Moderate | Ref. | Ref. |
|  | 3. Moderate-to-Severe | 1.70 (1.60, 1.82) | 1.34 (1.25, 1.45)^‡^ |
|  | 4. Severe | 2.65 (2.48, 2.84) | 1.94 (1.64, 2.29)^‡^ |
| **Atopic** | Yes | NR | 1.17 (1.02, 1.34)^*^ |
|  | No | NR | Ref. |

*p<0.05; †p<0.001; ‡p<0.0001.

^a^Multivariable proportional hazards model with the following covariates: exacerbation type, gender, GINA Step, Leidy category;

^b^Multivariable proportional hazards model with the following covariates: exacerbation type, gender, BTS Step, atopy, Leidy category and exacerbation history.

BTS, British Thoracic Society; CI, confidence interval; GINA, Global Initiative for Asthma; HR, hazard ratio; NR, not referenced.

### Table S4. Proportion of patients with an asthma-related ED/hospital re-admission following an asthma-related exacerbation

|  | | **US database**  **(N=5,167)**  **N (%)** | | | **UK database**  **(N=2,904)**  **N (%)** | | |
| --- | --- | --- | --- | --- | --- | --- | --- |
|  |  | **30-days** | **60-days** | **90-days** | **30-days** | **60-days** | **90-days** |
| **Patients with re-admission** | | n=477 | n=645 | n=799 | n=135 | n=175 | n=203 |
| **Age group, years** | 12–17 | 79 (7.4) | 106 (9.9) | 129 (12.1) | 19 (5.1) | 26 (7.0) | 29 (7.8) |
|  | 18–34 | 153 (9.9) | 214 (13.9) | 275 (17.8) | 33 (5.1) | 38 (5.8) | 47 (7.2) |
|  | 35–44 | 116 (11.0) | 154 (14.5) | 192 (18.1) | 29 (5.3) | 37 (6.8) | 42 (7.7) |
|  | 45–54 | 91 (9.9) | 120 (13.1) | 143 (15.6) | 26 (5.2) | 34 (6.8) | 37 (7.4) |
|  | 55–64 | 35 (7.2) | 47 (9.7) | 55 (11.3) | 15 (4.3) | 21 (6.0) | 23 (6.6) |
|  | 65+ | 3 (3.3) | 4 (4.4) | 5 (5.4) | - | - | - |
| **Gender** | Female | 326 (9.5) | 425 (12.3) | 517 (15.0) | 91 (4.9) | 119 (6.4) | 140 (7.5) |
|  | Male | 151 (8.8) | 220 (12.8) | 282 (16.4) | 44 (4.3) | 56 (5.4) | 63 (6.1) |
| **Leidy category** | 1. Low | 101 (8.7) | 130 (11.1) | 150 (12.9) | 15 (3.1) | 20 (4.1) | 23 (4.7) |
|  | 2. Low-to-Moderate | 151 (7.4) | 196 (9.5) | 246 (12.0) | 42 (3.4) | 56 (4.5) | 68 (5.5) |
|  | 3. Moderate-to-Severe | 111 (9.2) | 159 (13.1) | 201 (16.6) | 72 (6.3) | 91 (8.0) | 103 (9.1) |
|  | 4. Severe | 114 (15.6) | 160 (21.8) | 202 (27.6) | 6 (13.6) | 8 (18.2) | 9 (20.5) |
| **Atopic** | Yes | - | - | - | 11 (7.2) | 13 (8.5) | 14 (9.2) |
|  | No | - | - | - | 124 (4.5) | 162 (5.9) | 189 (6.9) |

ED, emergency department.

### Table S5. Proportion of patients with an all-cause ED/hospital re-admission following an asthma-related exacerbation

|  | | **US database**  **(N=5,167)**  **N (%)** | | | **UK database**  **(N=2,904)**  **N (%)** | | |
| --- | --- | --- | --- | --- | --- | --- | --- |
|  |  | **30-days** | **60-days** | **90-days** | **30-days** | **60-days** | **90-days** |
| **Patients with re-admission** | | n=1,168 | n=1,913 | n=2,583 | n=554 | n=709 | n=829 |
| **Age group, years** | 12–17 | 196 (18.3) | 308 (28.8) | 421 (39.4) | 74 (20.0) | 93 (25.1) | 105 (28.4) |
|  | 18–34 | 385 (25.0) | 656 (42.5) | 881 (57.1) | 110 (16.9) | 155 (23.8) | 184 (28.3) |
|  | 35–44 | 269 (25.4) | 423 (39.9) | 581 (54.9) | 106 (19.5) | 129 (23.7) | 153 (28.1) |
|  | 45–54 | 219 (23.9) | 360 (39.3) | 476 (51.9) | 101 (20.2) | 128 (25.6) | 144 (28.8) |
|  | 55–64 | 89 (18.3) | 143 (29.4) | 188 (38.6) | 68 (19.5) | 84 (24.1) | 98 (28.1) |
|  | 65+ | 10 (10.9) | 23 (25.0) | 36 (39.1) | - | - | - |
| **Gender** | Female | 785 (22.8) | 1,264 (36.7) | 1,689 (49.0) | 378 (20.2) | 491 (26.2) | 572 (30.6) |
|  | Male | 383 (22.2) | 649 (37.7) | 894 (51.9) | 176 (17.0) | 218 (21.1) | 257 (24.9) |
| **Leidy category** | 1. Low | 269 (23.1) | 452 (38.7) | 586 (50.2) | 60 (12.3) | 77 (15.8) | 103 (21.1) |
|  | 2. Low-to-Moderate | 420 (20.4) | 664 (32.3) | 912 (44.4) | 203 (16.4) | 265 (21.4) | 309 (25.0) |
|  | 3. Moderate-to-Severe | 271 (22.4) | 449 (37.1) | 598 (49.3) | 274 (24.1) | 347 (30.6) | 397 (35.0) |
|  | 4. Severe | 208 (28.4) | 348 (47.5) | 487 (66.4) | 17 (38.6) | 20 (45.5) | 20 (45.5) |
| **Atopic** | Yes | - | - | - | 42 (27.5) | 47 (30.7) | 48 (31.4) |
|  | No | - | - | - | 512 (18.6) | 662 (24.1) | 781 (28.4) |

ED, emergency department.

**Table S6.** Frequency of total HRU in the 30 days after an exacerbation

|  |  | **US database** | **UK database** |
| --- | --- | --- | --- |
|  |  | N=27,865 | N=17,785 |
|  |  | **mean (SD)** | **mean (SD)** |
| **Overall** |  | 1.35 (0.75) | 2.49 (1.83) |
| **Age group, years** | 12–17 | 1.32 (0.76) | 2.20 (1.63) |
|  | 18–34 | 1.32 (0.77) | 2.44 (1.87) |
|  | 35–44 | 1.36 (0.71) | 2.50 (1.89) |
|  | 45–54 | 1.37 (0.74) | 2.46 (1.78) |
|  | 55–64 | 1.36 (0.75) | 2.49 (1.81) |
|  | 65+ | 1.36 (0.79) | - |
| **Gender** | Female | 1.36 (0.77) | 2.54 (1.86) |
|  | Male | 1.33 (0.70) | 2.36 (1.76) |
| **GINA Step** | Step 1 | 1.28 (0.68) | 2.37 (1.70) |
|  | Step 2 | 1.35 (0.78) | 2.28 (1.71) |
|  | Step 3 | 1.30 (0.69) | 2.37 (1.72) |
|  | Step 4 | 1.33 (0.70) | 2.48 (1.76) |
|  | Step 5 | 1.59 (0.92) | 2.75 (2.07) |
|  | Not classifiable/none | 1.20 (0.45) | 2.07 (1.45) |
| **Leidy category** | 1. Low | 1.31 (0.71) | 2.29 (1.65) |
|  | 2. Low-to-Moderate | 1.31 (0.69) | 2.39 (1.70) |
|  | 3. Moderate-to-Severe | 1.33 (0.74) | 2.60 (1.92) |
|  | 4. Severe | 1.47 (0.86) | 2.96 (2.41) |
| **Exacerbations during the  12-month pre-index period** | <2 | 1.31 (0.72) | 2.41 (1.74) |
|  | ≥2 | 1.51 (0.85) | 2.87 (2.14) |
| **Severe uncontrolled asthma^a^** | Yes | 1.58 (0.90) | 2.94 (2.20) |
|  | No | 1.32 (0.73) | 2.42 (1.75) |
| **Severe uncontrolled eosinophilic asthma^b^** | Yes | 1.79 (1.07) | 2.98 (1.93) |
|  | No | 1.44 (0.86) | 2.72 (2.01) |
| **Atopic** | Yes | 1.41 (0.80) | 2.59 (1.90) |
|  | No | 1.30 (0.70) | 2.48 (1.82) |
| **Season of exacerbation occurrence** | Spring | 1.33 (0.72) | 2.48 (1.78) |
|  | Summer | 1.38 (0.74) | 2.49 (1.86) |
|  | Fall | 1.35 (0.76) | 2.53 (1.89) |
|  | Winter | 1.34 (0.76) | 2.46 (1.78) |

^a^Defined as patients at GINA Step 4 or 5 with ≥2 exacerbations in the previous 12 months; ^b^patients with severe uncontrolled asthma criteria and blood eosinophil counts ≥300 cells/µl at baseline.
GINA, Global Initiative for Asthma; HRU, healthcare resource utilization, SD, standard deviation.

**Table S7.** Medication utilization in the 30 days after an exacerbation (non-mutually exclusive categories)

|  |  |  | | **Asthma medication during 30-day period following an exacerbation  (non-mutually exclusive categories)^a^** | | | | | | | | | | | | | | |
| --- | --- | --- | --- | --- | --- | --- | --- | --- | --- | --- | --- | --- | --- | --- | --- | --- | --- | --- |
|  |  | **Number of exacerbations** | | | **SABA**  **n (%)** | | **LTRA**  **n (%)** | | **ICS**  **n (%)** | | **ICS/LABA**  **n (%)** | | **OCS**  **n (%)** | | **Anti-IgE**  **n (%)** | | **Theophylline^b^**  **n (%)** | |
|  |  | **US** | **UK** | | **US** | **UK** | **US** | **UK** | **US** | **UK** | **US** | **UK** | **US** | **UK** | **US** | **UK** | **US** | **UK** |
| **Overall** |  | 35,780 | 23,331 | | 8,017 (22.4) | 9,770  (41.9) | 5,498 (15.4) | 2,611  (11.2) | 2,639 (7.4) | 2,547  (10.9) | 5,590 (15.6) | 8,221  (35.2) | 4,150 (11.6) | 4,367  (18.7) | 467 (1.3) | N/A | 197  (0.6) | 957  (4.1) |
| **Age group, years** | 12–17 | 6,162 | 1,976 | | 1,332 (21.6) | 801  (40.5) | 823 (13.4) | 272  (13.8) | 590  (9.6) | 222  (11.2) | 637 (10.3) | 464  (23.5) | 521  (8.5) | 232  (11.7) | 48  (0.8) | N/A | 2  (0) | 47  (2.4) |
|  | 18–34 | 7,612 | 4,097 | | 1,789 (23.5) | 1,598  (39.0) | 814 (10.7) | 352  (8.6) | 445  (5.8) | 344  (8.4) | 888 (11.7) | 1,157  (28.42) | 793 (10.4) | 540  (13.2) | 62  (0.8) | N/A | 13  (0.2) | 118  (2.9) |
|  | 35–44 | 7,391 | 4,148 | | 1,673 (22.6) | 1,767  (42.6) | 1,092 (14.8) | 531  (12.8) | 459  (6.2) | 399  (9.6) | 1,164 (15.7) | 1,414  (34.1) | 930 (12.6) | 718  (17.3) | 96  (1.3) | N/A | 42  (0.6) | 229  (5.5) |
|  | 45–54 | 8,065 | 4,538 | | 1,852 (23.0) | 1,970  (43.4) | 1,505 (18.7) | 579  (12.8) | 593  (7.4) | 505  (11.1) | 1,598 (19.8) | 1,669  (36.8) | 1,081 (13.4) | 845  (18.6) | 163  (2.0) | N/A | 65  (0.8) | 222  (4.9) |
|  | 55–64 | 5,547 | 3,666 | | 1,150 (20.7) | 1,536  (41.9) | 1,069 (19.3) | 419  (11.4) | 464  (8.4) | 431  (11.8) | 1,105 (19.9) | 1,533  (41.8) | 700 (12.6) | 762  (20.8) | 90  (1.6) | N/A | 60  (1.1) | 141  (3.9) |
|  | 65+ | 1,003 | 4,906 | | 221  (22) | 2,098 (42.8) | 195 (19.4) | 458  (9.3) | 88  (8.8) | 646 (13.2) | 198 (19.7) | 1,984 (40.4) | 125 (12.5) | 1,270 (25.9) | 8  (0.8) | N/A | 15  (1.5) | 200  (4.1) |
| **Gender** | Female | 23,027 | 15,739 | | 5,073 (22.0) | 6,603  (42.0) | 3,748 (16.3) | 1,816  (11.5) | 1,698 (7.4) | 1,730  (11.0) | 3,578 (15.5) | 5,658  (36.0) | 2,740 (11.9) | 3,045  (19.4) | 295  (1.3) | N/A | 144  (0.6) | 718  (4.6) |
|  | Male | 12,753 | 7,592 | | 2,944 (23.1) | 3,167  (41.7) | 1,750 (13.7) | 795  (10.5) | 941  (7.4) | 817 (10.8) | 2,012 (15.8) | 2,563 (33.8) | 1,410 (11.1) | 1,322  (17.4) | 172  (1.3) | N/A | 53  (0.4) | 239 (3.2) |
| **GINA Step** | Not classifiable | 54 | 63 | | 8  (14.8) | 21  (33.3) | 3  (5.6) | 0 | 3  (5.6) | 12  (19.1) | 4  (7.4) | 11  (17.5) | 6  (11.1) | 13  (20.6) | 0 | N/A | 17  (31.5) | 4  (6.4) |
|  | Step 1 | 14,381 | 2,465 | | 3,207 (22.3) | 778  (31.6) | 773  (5.4) | 57  (2.3) | 820  (5.7) | 302  (12.3) | 1,050 (7.3) | 359  (14.6) | 1,534 (10.7) | 334  (13.6) | 16  (0.1) | N/A | 37  (0.3) | 46  (1.9) |
|  | Step 2 | 5,233 | 2,475 | | 1,050 (20.1) | 796  (32.1) | 1,632 (31.2) | 131  (5.3) | 442  (8.4) | 546  (22.1) | 464  (8.9) | 351  (14.2) | 617 (11.8) | 313  (12.7) | 18  (0.3) | N/A | 33  (0.6) | 12  (0.5) |
|  | Step 3 | 5,888 | 6,812 | | 1,250 (21.2) | 2,380  (34.9) | 624 (10.6) | 287  (4.2) | 819 (13.9) | 1,172  (17.2) | 846 (14.4) | 1,657  (24.3) | 602 (10.2) | 913  (13.4) | 12  (0.2) | N/A | 11  (0.2) | 43  (0.6) |
|  | Step 4 | 6,702 | 6,232 | | 1,567 (23.4) | 2,946  (47.3) | 1,599 (23.9) | 965  (15.5) | 363  (5.4) | 241  (3.9) | 2,091 (31.2) | 3,086  (49.5) | 757 (11.3) | 985  (15.8) | 53  (0.8) | N/A | 55  (0.8) | 293  (4.7) |
|  | Step 5 | 3,522 | 5,284 | | 935 (26.5) | 2,849  (53.9) | 867 (24.6) | 1,171  (22.2) | 192  (5.5) | 274  (5.2) | 1,135 (32.2) | 2,757  (52.2) | 634  (18.0) | 1,809  (34.2) | 368 (10.4) | N/A | 44  (1.2) | 559 (10.6) |
| **Leidy category** | 1. Low | 8,276 | 3,381 | | 1,154 (13.9) | 623  (18.4) | 1,016 (12.3) | 181  (5.4) | 573  (6.9) | 348  (10.3) | 1,039 (12.6) | 824  (24.4) | 620  (7.5) | 393  (11.6) | 52  (0.6) | N/A | 23  (0.3) | 21  (0.6) |
|  | 2. Low-to-Moderate | 13,654 | 9,313 | | 2,539 (18.6) | 2,604  (28.0) | 1,799 (13.2) | 690  (7.4) | 1,029 (7.5) | 1,005  (10.8) | 1,836 (13.4) | 2,615  (28.1) | 1,204 (8.8) | 1,271  (13.7) | 80  (0.6) | N/A | 33  (0.2) | 178  (1.9) |
|  | 3. Moderate-to-Severe | 7,680 | 9,949 | | 2,335 (30.4) | 6,269  (63.0) | 1,360 (17.7) | 1,593  (16.0) | 581  (7.6) | 1,127  (11.3) | 1,380 (18.0) | 4,480  (45.0) | 966 (12.6) | 2,471  (24.8) | 89  (1.2) | N/A | 61  (0.8) | 710  (7.1) |
|  | 4. Severe | 6,170 | 688 | | 1,989 (32.2) | 274  (39.8) | 1,323 (21.4) | 147  (21.4) | 456  (7.4) | 67  (9.7) | 1,335 (21.6) | 302  (43.9) | 1,360 (22.0) | 232  (33.7) | 246  (4.0) | N/A | 80  (1.3) | 48  (7.0) |
| **Exacerbations during the  12-month pre-index period** | <2 | 31,346 | 19,695 | | 6,589 (21.0) | 7,670  (38.9) | 4,593 (14.7) | 1,809  (9.2) | 2,324 (7.4) | 2,244  (11.4) | 4,656 (14.9) | 6,584  (33.4) | 3,265 (10.4) | 3,136  (33.9) | 255  (0.8) | N/A | 146  (0.5) | 523  (2.7) |
|  | ≥2 | 4,434 | 3,636 | | 1,428 (32.2) | 2,100  (57.8) | 905 (20.4) | 802  (22.1) | 315  (7.1) | 303  (8.3) | 934 (21.1) | 1,637  (45.0) | 885  (20.0) | 1,231  (33.9) | 212  (4.8) | N/A | 51  (1.2) | 434 (11.9) |
| **Severe uncontrolled asthma^c^,** | Yes | 2,091 | 2,782 | | 658 (31.5) | 1,702  (61.2) | 562 (26.9) | 742  (26.7) | 128  (6.1) | 164  (5.9) | 686 (32.8) | 1,425  (51.2) | 444 (21.2) | 1,010  (36.3) | 197  (9.4) | N/A | 35  (1.7) | 410 (14.7) |
|  | No | 33,689 | 20,549 | | 7,359 (21.8) | 8,068  (39.3) | 4,936 (14.7) | 1,869  (9.1) | 2,511 (7.5) | 2,383  (11.6) | 4,904 (14.6) | 6,796  (33.1) | 3,706 (11.0) | 3,357  (16.3) | 270  (0.8) | N/A | 162  (0.5) | 547  (2.7) |
| **Severe uncontrolled eosinophilic asthma^d^,** | Yes | 132 | 497 | | 58  (43.9) | 295  (59.4) | 32  (24.2) | 129  (26.0) | 9  (6.8) | 22  (4.4) | 43  (32.6) | 248  (49.9) | 41  (31.1) | 215  (43.3) | 9  (6.8) | N/A | 0 | 86  (17.3) |
|  | No | 3,855 | 6,622 | | 783 (20.3) | 2,802  (42.3) | 683 (17.7) | 704 (10.6) | 235  (6.1) | 788  (11.9) | 613 (15.9) | 2,486  (37.5) | 523 (13.6) | 1,418  (21.4) | 65  (1.7) | N/A | 34  (0.9) | 266  (4.0) |
| **Atopy status** | Yes | 14,193 | 1,314 | | 2,932 (20.7) | 598 (45.5) | 2,771 (19.5) | 204 (15.5) | 1,072 (7.6) | 156 (11.9) | 2,484 (17.5) | 482 (36.7) | 1,711 (12.1) | 240 (18.3) | 345  (2.4) | N/A | 78  (0.5) | 56  (4.3) |
|  | No | 21,587 | 22,017 | | 5,085 (23.6) | 9,172 (41.7) | 2,727 (12.6) | 2,407 (10.9) | 1,567 (7.3) | 2,391 (10.9) | 3,106 (14.4) | 7,739 (35.2) | 2,439 (11.3) | 4,127 (18.7) | 122  (0.6) | N/A | 119  (0.6) | 901  (4.1) |
| **Season of exacerbation occurrence** | Spring | 9,929 | 5,572 | | 2,095 (21.1) | 2,329 (41.8) | 1,588 (16.0) | 632 (11.3) | 774 (7.8) | 615 (11.0) | 1,550 (15.6) | 2,038 (36.6) | 1,049 (10.6) | 1,009 (18.1) | 124  (1.2) | N/A | 57  (0.6) | 253  (4.5) |
|  | Summer | 6,290 | 4,522 | | 1,440 (22.9) | 1,910 (42.2) | 1,041 (16.6) | 568 (12.6) | 469 (7.5) | 467 (10.3) | 1,092 (17.4) | 1,614 (35.7) | 768 (12.2) | 883 (19.5) | 106  (1.7) | N/A | 43  (0.7) | 189  (4.2) |
|  | Fall | 9,046 | 6,430 | | 2,230 (24.7) | 2,789 (43.4) | 1,382 (15.3) | 720 (11.2) | 687 (7.6) | 721 (11.2) | 1,343 (14.8) | 2,258 (35.1) | 1,119 (12.4) | 1,303 (20.3) | 122  (1.3) | N/A | 52  (0.6) | 253  (3.9) |
|  | Winter | 10,515 | 6,807 | | 2,252 (21.4) | 2,742 (40.3) | 1,487 (14.1) | 691 (10.2) | 709 (6.7) | 744 (10.9) | 1,605 (15.3) | 2,311 (34.0) | 1,214 (11.5) | 1,172 (17.2) | 115  (1.1) | N/A | 45  (0.4) | 262  (3.9) |

^a^Percentages are calculated as a percentage of the total number of exacerbations per category; ^b^including theophylline derivatives; ^c^defined as patients at GINA Step 4 or 5 with ≥2 exacerbations in the previous 12 months; ^d^patients with severe uncontrolled asthma criteria and blood eosinophil counts
≥300 cells/µl at baseline.

GINA, Global Initiative for Asthma; ICS, inhaled corticosteroid; IgE, immunoglobulin E; LABA, long-acting β_2_-agonist; LTRA, leukotriene receptor antagonist; N/A, not applicable; OCS, oral corticosteroid; SABA, short-acting β_2_-agonist.

**Table S8.** Mean healthcare cost per exacerbation in the 30 days after an exacerbation (US database only)

|  |  | **Total all-cause ($)** | **Total asthma-related ($)** |
| --- | --- | --- | --- |
|  |  | **mean (SD)** | **mean (SD)** |
| **Age group, years** | 12–17 | 1,002 (3,560) | 498 (921) |
|  | 18–34 | 1,235 (3,133) | 515 (954) |
|  | 35–44 | 1,384 (3,566) | 545 (1,060) |
|  | 45–54 | 1,532 (4,005) | 558 (1,093) |
|  | 55–64 | 1,600 (4,626) | 476 (834) |
|  | 65+ | 1,456 (3,561) | 442 (687) |
| **Gender** | Female | 1,410 (3,776) | 521 (1,007) |
|  | Male | 1,287 (3,837) | 517 (932) |
| **Leidy category** | 1. Low | 1,292 (4,302) | 477 (886) |
|  | 2. Low-to-Moderate | 1,257 (3,477) | 461 (783) |
|  | 3. Moderate-to-Severe | 1,321 (3,600) | 494 (947) |
|  | 4. Severe | 1,742 (3,953) | 687 (1,322) |
| **Atopy status** | Yes | 1,382 (3,430) | 546 (1,003) |
|  | No | 1,359 (4,033) | 501 (965) |
| **Season of exacerbation occurrence** | Spring | 1,356 (3,827) | 518 (971) |
|  | Summer | 1,441 (4,076) | 553 (1,057) |
|  | Fall | 1,343 (3,316) | 524 (968) |
|  | Winter | 1,357 (3,984) | 497 (952) |

SD, standard deviation.

**Table S9.** Mean healthcare cost per exacerbation in the 30 days after an exacerbation (US only)

|  |  | **All-cause ($)** | | **Asthma-related ($)** | | | | |
| --- | --- | --- | --- | --- | --- | --- | --- | --- |
|  |  | **Medical** | **Pharmacy** | **ED/hospital admission** | **Inpatient** | **ED** | **Outpatient** | **Pharmacy** |
|  |  | **mean (SD)** | **mean (SD)** | **mean (SD)** | **mean (SD)** | **mean (SD)** | **mean (SD)** | **mean (SD)** |
| **Overall** |  | 1,329 (4,117) | 361 (604) | 995 (1,652) | 5,227 (3,948) | 791 (1,047) | 344 (750) | 261 (327) |
| **Age group, years** | 12–17 | 950 (3,837) | 280 (611) | 831 (1,440) | 4,570 (4,031) | 622 (561) | 308 (611) | 225 (266) |
|  | 18–34 | 1,307 (3,414) | 249 (384) | 915 (1,418) | 5,279 (4,259) | 746 (764) | 309 (615) | 206 (236) |
|  | 35–44 | 1,388 (3,859) | 334 (575) | 1,105 (1,792) | 5,428 (3,416) | 869 (1,227) | 365 (818) | 247 (258) |
|  | 45–54 | 1,472 (4361) | 422 (688) | 1,287 (2,094) | 5,635 (4,134) | 1,001 (1,470) | 379 (818) | 303 (446) |
|  | 55–64 | 1,470 (5,023) | 467 (652) | 857 (1,484) | 6,312 (4,741) | 762 (1,187) | 376(889) | 298(303) |
|  | 65+ | 1,222 (3796) | 501 (681) | 829 (1,429) | 3,131 - | 782 (1,405) | 233 (405) | 330 (413) |
| **Gender** | Female | 1,360 (4,069) | 356 (553) | 1,034 (1,786) | 5,615 (4,053) | 811 (1,151) | 338 (730) | 257 (262) |
|  | Male | 1,264 (4,212) | 371 (696) | 920 (1,359) | 4,477 (3,662) | 752 (810) | 358 (789) | 269 (422) |
| **GINA Step** | Step 1 | 1,305 (4,315) | 260 (519) | 913 (1,492) | 4,984 (3,838) | 767 (1,062) | 241 (424) | 168 (186) |
|  | Step 2 | 1,281 (4,317) | 395 (564) | 1,033 (1,772) | 5,169 (4,674) | 768 (833) | 283 (507) | 247 (224) |
|  | Step 3 | 1,234 (3,537) | 322 (465) | 941 (1,632) | 5,835 (3,330) | 724 (1,015) | 254 (406) | 242 (227) |
|  | Step 4 | 1,324 (4,188) | 458 (716) | 1,002 (1,521) | 5,195 (3,549) | 759 (793) | 282 (563) | 337 (383) |
|  | Step 5 | 1,644 (3,799) | 528 (783) | 1,515 (2,404) | 5,428 (4,592) | 1,166 (1,614) | 870 (1,537) | 379 (547) |
|  | Not Classifiable | 819 (1,732) | 328 (405) | 477 (414) | 0 | 477 (414) | 111 (89) | 247 (337) |
| **Leidy category** | 1. Low | 1,292 (4,725) | 321 (521) | 859 (1,493) | 4,110 (3,432) | 762(1,280) | 283 (618) | 250 (262) |
|  | 2. Low-to-Moderate | 1,214 (3,735) | 331 (552) | 829 (1,223) | 4,290 (3,217) | 691 (765) | 290 (598) | 244 (260) |
|  | 3. Moderate-to-Severe | 1,297 (3,953) | 355 (606) | 1,010 (1,739) | 6,538 (4,828) | 781 (872) | 325 (688) | 247 (269) |
|  | 4. Severe | 1,643 (4,225) | 469 (748) | 1,550 (2,339) | 5,804 (3,964) | 1,084 (1,370) | 513 (1,063) | 313 (485) |
| **Exacerbations during the  12-month pre-index period** | <2 | 1,308 (4,167) | 354 (594) | 914 (1,426) | 4,621 (3,291) | 773 (1,060) | 309 (686) | 254 (280) |
|  | ≥2 | 1,469 (3,758) | 405 (658) | 1,336 (2,351) | 6,362 (4,785) | 868 (988) | 522 (997) | 302 (510) |
| **Severe uncontrolled asthma^a^** | Yes | 1,543 (3,396) | 507 (757) | 1,599 (2,521) | 6,034 (4,496) | 1,079 (1,298) | 700 (1,209) | 392(655) |
|  | No | 1,314 (4,161) | 351 (590) | 946 (1,550) | 5,066 (3,831) | 768 (1,021) | 310 (679) | 249 (276) |
| **Severe uncontrolled eosinophilic asthma^b^** | Yes | 1,881 (3,516) | 498 (522) | 2,662 (3,540) | 5,195 (4,431) | 1,739 (1,867) | 726 (1,265) | 294 (270) |
|  | No | 1,594 (4,191) | 430 (650) | 1,296 (2,165) | 5,074 (4,011) | 1,002 (1,589) | 419 (921) | 261 (268) |
| **Atopic** | Yes | 1,252 (3,632) | 404 (663) | 848 (1,524) | 5,514 (4,365) | 675 (930) | 434 (942) | 295 (404) |
|  | No | 1,386 (4,440) | 331 (557) | 1,072 (1,710) | 5,120 (3,798) | 852 (1,099) | 276 (553) | 237 (254) |
| **Season of exacerbation occurrence** | Spring | 1,307 (4,149) | 364 (613) | 976 (1,597) | 5,221 (4,426) | 810 (1,040) | 338 (739) | 266 (364) |
|  | Summer | 1,398 (4,409) | 375 (607) | 976 (1,767) | 5,681 (4,187) | 725 (1,001) | 373 (850) | 283 (370) |
|  | Fall | 1,310 (3,564) | 356 (648) | 1,039 (1,613) | 5,114 (3,548) | 809 (963) | 344 (708) | 251 (307) |
|  | Winter | 1,323 (4,346) | 354 (548) | 984 (1,662) | 4,997 (3,925) | 801 (1,160) | 333 (728) | 252 (273) |

^a^Defined as patients at GINA Step 4 or 5 with ≥2 exacerbations in the previous 12 months; ^b^patients with severe uncontrolled asthma criteria and blood eosinophil counts ≥300 cells/µl at baseline.

ED, emergency department; GINA, Global Initiative for Asthma; SD, standard deviation.
